# How Neoliberalism Shapes Indigenous Oral Health Inequalities Globally: Examples from Five Countries

**DOI:** 10.3390/ijerph17238908

**Published:** 2020-11-30

**Authors:** Lisa Jamieson, Joanne Hedges, Sheri McKinstry, Pauline Koopu, Kamilla Venner

**Affiliations:** 1 Indigenous Oral Health Unit, University of Adelaide Dental School, Adelaide 5005, Australia; joanne.hedges@adelaide.edu.au; 2 College of Dentistry, University of Saskatchewan, E3350-107 Wiggins Road, Saskatoon, SK S7N 5E4, Canada; sheri.mckinstry@usask.ca; 3 Auckland Regional Hospital & Specialist Dentistry, Auckland District Health Board, 1023 Auckland, New Zealand; paulinek@adhb.govt.nz; 4 Center on Alcoholism, Department of Psychology, Substance Abuse, & Addiction, University of New Mexico, Albuquerque, NM 87131, USA; kamilla@unm.edu

**Keywords:** indigenous, neoliberalism, oral health, Māori, aboriginal, Torres Strait Islander, Sámi, Alaskan Native, Native American, First Nations, Inuit, Métis

## Abstract

Evidence suggests that countries with neoliberal political and economic philosophical underpinnings have greater health inequalities compared to less neoliberal countries. But few studies examine how neoliberalism specifically impacts health inequalities involving highly vulnerable populations, such as Indigenous groups. Even fewer take this perspective from an oral health viewpoint. From a lens of indigenous groups in five countries (the United States, Canada, Australia, Aotearoa/New Zealand and Norway), this commentary provides critical insights of how neoliberalism, in domains including colonialism, racism, inter-generational trauma and health service provision, shapes oral health inequalities among Indigenous societies at a global level. We posit that all socially marginalised groups are disadvantaged under neoliberalism agendas, but that this is amplified among Indigenous groups because of ongoing legacies of colonialism, institutional racism and intergenerational trauma.

## 1. Introduction

Neoliberalism, a political orientation influenced by economists Friedman and Hayek, is the dominant economic and philosophical model underpinning the operation of many Organisation for Economic Co-operation and Development (OECD) governments [1]. It is characterised by markets that are competitive and private, reduced public expenditure on infrastructure and social services, and deregulation that better facilitates freedom of choice and economic activity [2]. Individual autonomy is strongly endorsed, particularly individual responsibility for health and wellbeing [3]. Champions of neoliberalism cite how millions have been saved from extreme poverty because of global trade’s expansion and how direct investment from foreign entities is a means of reallocating both know-how and technology to countries with economies that are still developing [4]. Privatization of state-owned enterprises has, in many cases, led to more efficient provision of services and lowered the fiscal burden on governments [5]. However, critics have described how neoliberal policies increase both economic and health inequality [6,7], with more today living in poverty than before neoliberalism [8].

Indigenous peoples, as defined by the United Nations Indigenous Peoples’ Partnership, includes those with a history of invasion and colonialism on their lands that are sustained to this day [9]. Identification is typically two-fold; both through an individual identifying as Indigenous and by recognition and acceptance by a given Indigenous population. This enables Indigenous peoples to act upon their sovereign right to, without interference from outside parties, make decisions regarding group membership [10]. At a global level, most Indigenous groups share similar challenges relating to protection of their rights, even though there are substantial cultural and geographic differences between such groups [11]. In the contemporary global setting, Indigenous peoples represent some of the most socially disadvantaged and alienated groups [12]. Almost without exception, strategic and sustained government policies of cultural annihilation, assimilation, discrimination and marginalization have impacted on most Indigenous peoples [13]. Understandingly, the impacts on Indigenous health of such discriminatory historical legacies, most of which violated the most fundamental human rights, have been immeasurable. However, only recently, and only in some countries, has there been both recognition and efforts made to redress these grievous inequities [14]. It is important to highlight that Indigenous peoples represent so much more than collectives of ‘socially disadvantaged’ populations [15]. The historical treatments of Indigenous groups is reflected contemporarily by the sometimes universal poverty and inequality experienced; this is not always the case with other members of society who are socially disenfranchised [16]. Because these historical and social determinants are unique, with Indigenous persons often being the only groups to experience them over such sustained periods, such special circumstances need to be considered when health outcomes of Indigenous groups are reported and discussed.

Oral health offers an exquisite mirror of social injustice. Indeed, some of the largest health inequalities documented at an international level involve oral health status [17]. This is because oral health is simultaneously a reflection of unequal material circumstances, unequal access to health services and structural inequities over the life course [18]. Measured objectively through clinical oral health examinations (for example, assessments of decayed, missing and filled teeth, periodontal disease, oral cancer) or subjectively (self-rated oral health, oral health-related quality of life, perceived need for dental care), oral health inequalities exist across all countries [19], all age groups [20] and all disease states [21]. Oral health inequalities among Indigenous groups in most countries are particularly high [22], with the possible exception of Norway. The little empirical evidence available from this country suggests that there is no or little difference between Indigenous Sámi and non-Indigenous Norwegians.

From a lens of Indigenous groups in five countries (the United States, Canada, Australia, Aotearoa/New Zealand and Norway), this commentary provides critical insights of how neoliberalism, in domains including colonialism, racism, inter-generational trauma and health service provision, shapes oral health inequalities among Indigenous societies at a global level. The authors chose these five countries because of consistencies in the respective histories of neoliberalism, impacts of colonialism on the Indigenous populations and health service provision models [23,24].

*Nature of the engagement, involvement and leadership by Indigenous people:* Four of the five authors identify as Indigenous and represent each of the countries included in the commentary (with the exception of Norway). All are recognised leaders in Indigenous rights and advocacy broadly, and in the provision of culturally-safe health care services specifically. 

*Whose priorities are being addressed by the study:* Priorities that are highlighted in the commentary reflect what is reported in the literature, but also reflect concerns shared by all authors, many of whom have lived experience of the types of oral health inequities described.

Ethics approval was not required due to the commentary nature of the work.

## 2. Main Argument of Commentary

### 2.1. United States

*Neoliberalism in the United States*: The foundational roots of neoliberalism in the United States were established in the 1970s during the Carter administration [25]. Initially, it involved the airline, trucking and banking sectors being deregulated [26]. Under the Reagan administration, in the 1980s, this trend intensified to include expansion of the trade deficit, deregulation of the financial sector, increased spending on defence and tax cuts [27]. In 1981, federal income taxes were substantially reduced by 25% [28]. Neoliberalism continued to be embraced during the Clinton administration, in the 1990s, with increased welfare state cuts, increased deregulation of the finance sector, and support of the North American Free Trade Agreement being supported [29]. In the contemporary setting, both major political parties in the US have embraced progressive neoliberalism, which American feminist philosopher Nancy Fraser describes as being a “progressive-neoliberal alliance of financialisation plus emancipation” [30]. 

*Indigenous Populations in the United States*: Historic boarding schools for American Indian or Alaskan Native (AI/AN) persons were established in the 1870s by European colonisers to detribalize and assimilate Indigenous peoples into Euro-American culture [31]. The schools were operated by Christian missionaries until the early 20th century, at which point the federal government assumed governance. Students could not speak their native language, were dressed in Euro-American styled clothing, and taught about Western civilization. The historic legacy of boarding schools for AI/ANs is that of trauma and abuse [32]. Contemporary AI/AN communities are critical of these past educational practices, but also of contemporary efforts that empower Euro-American pedagogy and minimize the validity of Indigenous education systems and pedagogy [33]. Today many tribal nations insist on community-based schools, including tribal colleges and universities.

Estimates from the US Census Bureau indicate that, in 2018, approximately 2.2% of the total US population identified as being AI/AN [34], with approximately 42% being aged 24 years or less. The largest populations are in New Mexico, Oklahoma, California and Arizona, with around 60% residing in urban communities. There are 574 culturally diverse and federally recognised tribes (229 in Alaska alone). Because each tribal group acts as its own government, tribes have substantial governance, decision-making and power, within their respective territories. This includes being able to manage and regulate health services. Because tribes have the ultimate sovereign power over their peoples, country and other related affairs, they are similar to federal and state governments. Each sovereign tribal nation also has, however, a relationship with the US government that is effectively government-to-government, with the US government being mandated to both protect and promote resources that are tribal. 

Although a relationship of trust with the US government, through federal policies, is meant to improve the AI/AN–US government relationship via the provision of health, education and other social services, the forced assimilation into mainstream America of AI/AN people from their traditional lands and customs due to past government initiatives, sustained poverty, historical and intergenerational trauma, and increasing inequalities in health, education and justice have resulted in AI/AN peoples having much higher rates of unemployment, incarceration, alcohol use disorders, substance misuse, poor mental health, chronic diseases, infections and overall mortality compared with non-AI/AN people [35,36]. Twenty five percent of AI/AN people live below the federal poverty line [37]. The proportion of AI/AN people on reservations living below poverty ranges from 38 to 53% % [37]. Among employed AI/AN people, a high proportion earn below-poverty wages [38].

*Dental Disease and Dental Health Service Provision for Indigenous Persons in the US*: Like almost all other health outcomes, dental disease levels among AI/AN populations are high. Over two-thirds (70%) of Navajo children, who comprise the second largest tribe in the United States, have teeth with untreated dental caries [39], around 64% of 35 to 49 year old AI/AN adults have untreated dental caries (compared with 27% of non-AI/AN Americans), and around 46% of AI/AN adults aged 65+ years have untreated dental caries compared with 19% of their non-AI/AN counterparts [40]. Around 17% of AI/AN adults aged 35+ years have severe periodontal disease compared with 10% of general United States adults [38].

The Indian Health Service (IHS)—which operates within the US Department of Health and Human Services—provides medical and other health care services to AI/AN people who are members of federally recognised tribes. This is means-tested and orchestrated through IHS clinics located specifically on tribal lands. Urban programs are also available, serving around 600,000 people (not all urban programs offer dental service provision however). Several tribes are actively involved in IHS program implementation, while others operate their own health systems independent of the IHS. The service at many, but not all, IHSs include provision of dental care. However, dental services provided by the IHS are severely underfunded, with 90% of its services being provision of basic, emergency care [41]. Preventive and restorative care is generally provided only to children. AI/AN people are consequently denied endodontic care, crowns, bridges, dentures and surgical extractions [42]. The IHS additionally struggles with geographic isolation of many tribal populations and difficulties attracting dentists to practice. This is despite intense recruitment efforts and substantial financial incentives. As Batliner described, however, even if all oral health-related positions serving AI/AN people were occupied, the ratio of oral health provider-to-population would still be low due to the overwhelming burden of untreated dental caries occurring in the population; a large proportion of whom are spread diffusely across large geographic areas [41].

### 2.2. Canada

*Neoliberalism in Canada*: A shift in the emphasis of monetary policy in Canada commenced in the early 1980s, with the Trudeau government (in 1982) leading a wide-ranging commission into Canada’s economic prospects that subsequently resulted in a highly neoliberal and globalised vision of economic policy [43]. Free trade was a key recommendation of the commission, which also included proposed domestic economic and social policies with a greater reliance on market forces. A comprehensive free trade agreement with the US was consequently established in 1988. Canadian government economic, social and fiscal policies began to mirror benchmarks established in the US in the mid-1990s. 

*Indigenous Populations in Canada*: The Indigenous peoples of Canada are those who identify as First Nations, Inuit and/or Métis (of mixed Indigenous and Euro-American ancestry). In the 2016 census, Indigenous peoples in Canada represented 4.9% of the total population, with almost half being aged 25 years or less. Around 56% of Indigenous Canadians reside in metropolitan locations, with the remainder living on reserves. In 2016, Indigenous Canadian children aged 0–14 years accounted for 7.7% of Canada’s total child population, with 52% of children in foster care being Indigenous (meaning approximately 4% of Indigenous children are in foster care) [44,45]. In Canada, more than 600 First Nations governments or bands are federally recognised, and over 70 different languages.

Under the guise of ‘cultural assimilation’, from 1847 to 1996, and in partnership with Christian churches, the Canadian government administered, throughout Canada, a system of residential boarding schools for Indigenous children, who were removed forcibly from their homes and families [46]. While the premise of the schools was education, there was widespread under-funding, disease and abuse [47]. While these schools essentially served as an assimilatory tool, the flawed rationalisation for originally removing First Nations children from their families and homes resulted in schools additionally acting as orphanages and child-welfare facilities for Indigenous children from the 1940s. This would set the stage for what is known today as the ‘Sixties Scoop’ (series of policies enacted by provincial child welfare authorities which saw thousands of Indigenous children taken from their families, placed in foster homes, and eventually adopted into White families) and the transition into the contemporary child-welfare system. Though there were few regulations, the Indian Act that defined and controlled First Nations in Canada was the crucial legislation governing these schools [48]. The Canadian government, in 1995, launched the ‘Aboriginal Right to Self-Government Policy’. For the first time in Canadian history, this policy acknowledges that both Inuit and First Nations have the right, which is constitutional, to form and govern their own government in accordance with First Nation and Inuit economic, political, cultural and historical circumstances. While the Federal Government does recognize this inherent right, not all communities/health regions self-govern, however.

*Dental Disease and Dental Health Service Provision for Indigenous Persons in Canada*: Indigenous Canadians experience poorer oral health than non-Indigenous Canadians. Using data from the 2007-09 Canadian Health Measures Survey, the prevalence of untreated dental caries among those identifying as First Nations, Métis or Inuit was 38%, compared with 19% among those identifying as being non-Indigenous; a two-fold difference [49]. Around 50% of Indigenous Canadians had missing teeth, compared with 44% of non-Indigenous Canadians. Almost one-third of First Nations adults experienced difficulties accessing dental care, and although 71% of First Nations children obtained dental treatment within a year of a First Nations national survey, 22% of three to five year-olds obtained their dental treatment more than 90 kilometres from their community [50].

The dental health care system in Canada is predominately financed privately. This is largely through benefit plans that are employment-based. Around 6% of the population (which includes a large proportion of Indigenous Canadians) is covered by public dental insurance [51]. First Nations persons who are registered under the Indian Act and Inuk who are recognized by an Inuit land claim organization are eligible for certain services such as dental care, by the Non-Insured Health Benefits (NIHB) program when medically necessary goods and services are not covered by other programs such as social programs, private insurance, or provincial/territorial health insurance [52].

A framework developed to better enable the assumption of health services control by Indigenous Canadians—The Indian Health Transfer Policy—has a strong focus on self-determination [53]. Through the Indian Health Transfer policy process, the decision to engage in discussions regarding potential transfer with Health Canada is the responsibility of each community. Once a transfer has been agreed upon, Indigenous communities assume control of all responsibilities of facilitating health programs at a pace set by each community’s health management capabilities and individual circumstances [54]. Many Indigenous Canadian health centres provide access to dental care, through provision of the First Nations and Inuit Health Branch. However, these programs are severely underfunded, with a chronic shortage of dental personnel to provide care and with a predetermination policy (where permission from Health Canada is required before treatment can commence) leading to delays to receipt of what is usually emergency treatment. As described by the Assembly of First Nations, the system of predetermination causes ‘prolongation of illness, lacks in compassion and adds considerable cost to the program, particularly if clients have travelled long distances’ [55]. Program utilization rates of Indigenous Canadian people are consequently far lower than rates for employer-sponsored plans (how the majority of Canadians access dental care). Some providers have opted out of the program, meaning patients have to pay out-of-pocket for dental care. This makes it impossible for a high proportion of First Nations and Inuit people to access dental care, due to the disproportionately low incomes of many. 

### 2.3. Australia

*Neoliberalism in Australia*: Neoliberal policies in Australia have been embraced by both major government parties (Labor and Liberal) since the 1980s. The Hawke and Keating governments (both Labor) from 1983 to 1996 set in train a large program of reform that emphasised economic liberalisation. This involved privatisation of government corporations, deregulation of markets, floating the Australian dollar and reducing trade protections [56]. In 1992, a compulsory superannuation system was implemented in order to limit future liability for the government for old age pensions. Financing of the university sector was deregulated, which meant students needed to pay university fees through a loan system. With increasing competition, this encouraged universities to change their business model to facilitate greater income by welcoming overseas students who would pay full fees [57]. 

*Indigenous Populations in Australia*: Indigenous Australians include those who identify as being Aboriginal and/or Torres Strait Islander. Indigenous Australians have resided in Australia for over 65,000 years, with their numbers decimated following introduced diseases and massacres resulting from colonisation in the 1780s. Contemporary Indigenous Australians represent 3.3% of the total Australian population [58]. At the time of European settlement, over 250 Indigenous Australian languages were spoken; around 145 of these remain in use, with 130 considered to be endangered. Early colonial ownership of land and water resources, which resulted in a rapid decline of kangaroo and other Indigenous foods, continued through the 19th and 20th centuries. In the modern setting, much of this traditional land has been converted into sheep and cattle grazing pastures [59]. This had profound impacts on the traditional nomadic, hunter/gatherer lifestyle of Indigenous Australians.

Throughout most of the 19th and 20th centuries, Indigenous Australians lived under the shadow of a range of state- and territory-based protection laws. Various Acts of Parliament approved both ‘Protectors of Aborigines’ and ‘Aboriginal Protection Boards’. The purpose of these roles was to ensure the apparent safety of Indigenous Australians, but in reality they served to control the lives and decisions of Indigenous Australians in all matters ranging from employment, marriage and childcare. The Protectors controlled wages, with many Indigenous Australians receiving no income, and others receiving less than their non-Indigenous counterparts in similar employment roles [60]. From 1810, Indigenous Australians were forcibly relocated to mission stations, run by churches and administered by the state. While providing food and shelter, the purpose of the missions was to primarily inculcate Western values. This period of protectionist policies that aimed to segregate and control Indigenous populations was rigorously challenged by those it sought to control. This resulted, in 1937, in the government moving towards assimilation policies. The purpose of these policies was to assimilate Indigenous Australians who were “not of full blood” into the non-Indigenous community, with the aim to remove the “Aboriginal problem”. Consequently, a large number of Indigenous children were forcibly removed from their homes and families, and placed in foster homes with white families or institutions [61]. The impacts of these ‘Stolen Generations’ tore at the very fabric of Indigenous Australian society and continues to have profound impacts on the social, emotional, educational, economic and physical wellbeing of Indigenous Australians today.

*Dental Disease and Dental Health Service Provision for Indigenous Persons in Australia*: Indigenous Australian children had better oral health than their non-Indigenous counterparts prior to the 1980s [62,63,64,65]. However, more recent evidence documents how Indigenous children have up to five times as much untreated dental caries than non-Indigenous children [66,67]. In a study of Indigenous children living in remote locations, more than 90% of dental disease experience comprised either decayed or missing teeth, with less than 10% of teeth with active dental decay having been filled [67]. Using data from the 2004-06 National Survey of Adult Oral Health, almost half (49%) of those identifying as Aboriginal and/or Torres Strait Islander had untreated dental caries, compared with less than one-quarter (23%) of those identifying as non-Aboriginal [68]. Around 79% of Aboriginal Australians had missing teeth, compared with 59% of non-Aboriginal Australians.

In Australia, dental care for adults is predominantly provided through the private sector. Provision of publically-funded dental care is available for those eligible through means-testing. This comprises a large proportion of Aboriginal Australians. There are long wait lists in the public dental sector, however, with most services requiring co-payments and a limited range of services. Health care for Indigenous Australians is predominantly provided through federally-funded Aboriginal Community Controlled Health Organisations (ACCHOs). Approximately one-third of ACCHOs provide dental services, but usually for a small out-of-pocket cost [69]. In the 2017-2018 National Survey of Adult Oral Health, more Indigenous Australians perceived a need for dental care, felt uncomfortable about their dental appearance, had experienced toothache in the last 12 months, avoided eating certain foods because of dental problems and delayed dental care due to cost than non-Indigenous Australians [70]. Substantial barriers persist in accessing dental care for Indigenous Australians, including experiences of racism in the dental setting, difficulties in making dental appointments, dental fear and having to travel substantial distances [69]. 

### 2.4. Aotearoa/New Zealand

*Neoliberalism in Aotearoa/New Zealand*: Arising from the knock-on effects of the Great Depression in the 1930s, Aotearoa/New Zealand legislated comprehensive welfare state policies in 1938. However, by the 1970s, political models embracing neoliberal frameworks including economic restructuring, increased market competition and privatisation, limited public expenditure on social services, and deregulation and promotion of individual responsibility were taking hold. By the mid-late 1980s, many producer subsidies and import tariffs were phased out, the financial market was deregulated and the exchange rate was floated. Severe cuts to welfare occurred in 1991, alongside wholesale health reform policies that aimed to, though a competitive market and privatization, expand opportunities for innovation and maximize efficiency [71]. The impacts of these reforms had severe consequences, with over 111,000 jobs lost between 1986 and 1996 [72]. In 2015, Aotearoa/New Zealand experienced one of the most substantial increases in economic inequality in the OECD since the 1980s; this in a country previously applauded for its relatively low income inequality [73].

*Indigenous Populations in Aotearoa/New Zealand*: Māori are the Indigenous people of Aotearoa/New Zealand, with the Māori language officially recognised as an Aotearoa/New Zealand national language. The Māori arrived via canoe from eastern Polynesia between 1320 and 1350. European colonisation, commencing in the 1780s, caused large changes to Māori way of life [74]. Initial Māori and European relationships were purportedly amicable, with the signing of the Treaty of Waitangi in 1840. However, land conflicts in the 1860s led to rising tensions and, ultimately, land wars. Social upheaval and epidemics from introduced diseases took an enormous toll on the Māori population [74]. In contemporary Aotearoa/New Zealand, where Māori represent 17% of the Aotearoa/New Zealand population (making them the second-largest ethnic group after European New Zealanders), a large number of Māori experience substantial social and economic challenges, and generally have lower incomes and life expectancies compared with other New Zealanders [75]. Māori New Zealanders suffer higher incarceration rates, health problems and poorer educational outcomes. The life expectancy gap between Māori and non-Māori populations in Aotearoa/New Zealand is 7 years [76]. 

*Dental Disease and Dental Health Service Provision for Indigenous Persons in Aotearoa/New Zealand*: Using data from the 2009 New Zealand Oral Health Survey, half (50%) of those identifying as Māori had untreated dental caries, compared with 34% of those identifying as non-Māori [77]. Almost two-thirds (65%) of the Māori population had missing teeth, compared with 46% of non-Māori people. Māori children had 1.5 times the rate of their non-Māori counterparts of having had teeth extracted because of untreated dental disease, abscesses or infection [77]. Aotearoa/New Zealand’s dental sector for adults is fundamentally private, with emergency dental services also provided through hospitals. Māori oral health providers operate under a government-funded and private business model. Dental care for children is provided free-of-charge, through the School Dental Service. Barriers to accessing timely and appropriate dental care among Māori have been identified as including cost, culturally safe care, service availability and dental fear [78].

### 2.5. Norway

*Neoliberalism in Norway*: Norway, like other Scandinavian countries (Sweden, Denmark, Finland and Iceland), has not embraced neoliberal ideologies as conclusively as many other OECD countries. The so-called ‘Nordic Model’ instead embraces income support and social services, including health, to be funded predominantly from taxation. The Nordic model’s origins are from the 1930s, with its foundations considered to be a compromise between worker and employer parties. Although social security and collective wage bargaining policies were reduced following economic imbalances and financial crises of the 1980s and 1990s, welfare expenditure remains high compared with the European and other OECD average [79]. The Nordic model, briefly, encapsulates the following: (1) a sophisticated social safety net, in addition to public services including universal health care and free education, in a system that is predominantly tax-funded; (2) free trade that is combined with collective risk sharing, which provide some protection against the inherent risks that economic openness is associated with [80]; (3) minimal produce market regulation; (4) high percentage of workers belonging to labour unions; (5) highest rankings on freedom from corruption, perceived freedom to make life choices, social support, life expectancy and gross domestic product (GDP) per capita. Norway was the highest ranked OECD country in the 2017 World Happiness Report [81], with Norway the only major economy in the West with a younger generation that is getting richer (13% increase in disposable income in 2018) [82]. 

The Norwegian state today has a multitude of ownership positions in sectors that include fresh water, seafood, timber, minerals, natural gas and petroleum. It also owns 37% of the Oslo stock market. One-quarter of the country’s GDP is accounted for by the petroleum industry alone [83].

*Indigenous Populations in Norway*: The Sámi people are the Indigenous Finno-Ugric group who inhabit large northern regions of Norway, Sweden, Finland and Russia’s Kola Peninsula. There is evidence of Sámi having inhabited this region for the last 3500 years [84]. Approximately half of the contemporary Sámi population reside in Norway; around 1.3% of total population. The Sámi are the only group acknowledged as being Indigenous in Norway, with the Sámi language officially recognised as a national minority language. Currently around 10% of Sámi in Norway are engaged in semi-nomadic reindeer herding as their livelihood [83]. Indeed, reindeer herding is legally reserved for only Sámi people in some regions of Norway, for political, cultural, environmental and traditional reasons [84]. 

Although the Sámi language and reindeer husbandry are still the cornerstone of contemporary Sámi culture (particularly in Finnmark, the most northern county of Norway, where the majority speak Sámi), most Sámi are in fact assimilated into Western ways of living. Historical processes of assimilation have, as with almost all other Indigenous cultures at a global level, not always been positive. For example, in the 18th and especially 19th centuries, the Sámi people were forced to speak Norwegian, with policies from the Norwegian state devaluing and restructuring Sámi culture and identity [85]. There was a brief period of eugenics in the 1930s [86]. From 1900 to 1940, the Norwegian state enacted a series of laws which meant that any person wanting to buy or lease state lands for agriculture in Finnmark had to both prove knowledge of the Norwegian language and register with a Norwegian name [87]. In 1913, the Norwegian parliament passed a Native Act Land bill to allocate the most useful lands to Norwegian settlers. Although this law has been relaxed, the legacy is still evident, for example, laws in the 1970s limiting the size of any house Sámi people were allowed to build [88]. In 1986, a national anthem and flag of the Sámi people were created. 

The Sámi population today is mostly urbanised, although a substantial proportion live in rural areas in the northernmost part of Norway [88]. In the past (50–60 years previous), Sámi people were coping with the intergenerational consequences of language and culture loss related to generations of Sámi children being removed from their families and placed in missionary and/or state-run boarding schools, as well as ongoing legacies of laws created to deny the Sámi rights of their beliefs, language, land and the practice of traditional livelihoods [89]. This is not the case for many contemporary Sámi, however, with all Sámi children having a legislated right to speak and to receive education in the Sámi language at school. Norway now constitutionally recognises two people; Norwegians and Sámi, with both having separate parliaments and official languages [90]. 

Compared with the profound inequalities experienced between Indigenous and non-Indigenous people globally, a lower magnitude of differences in life expectancy, health, nutrition and socioeconomic status have been reported between the Sámi in Norway and their non-Indigenous counterparts [91,92,93]. There are only minor differences in the health and wellbeing of Sámi and Norwegian peoples [94]. For example, researchers have reported negligible differences in the prevalence of central obesity and diet-related chronic conditions such as type 2 diabetes [95,96,97,98]. There is some empirical evidence of ethnic discrimination being experienced by the Sámi, with implications on self-reported health [90] and health outcomes including cardiovascular disease, diabetes, chronic muscle pain, metabolic syndrome and obesity [99], and psychological distress [100].

*Dental Disease and Dental Health Service Provision for Indigenous Persons in Norway*: In Norway’s Dental Health in the North Study, there were no reported differences in the prevalence of periodontal disease between Sámi and non-Sámi (50% vs. 49%). However, the Sámi had a higher prevalence of deep periodontal pockets and a higher probability of having more severe stages of periodontal disease compared to non-Sámi [101]. Sámi participants had a lower prevalence of dental caries compared with non-Sámi in the same area. This is likely due to Sami in this county living largely traditional lifestyles with less exposure to cariogenic foods and beverages. Teterina and colleagues found that, when comparing Sámi core areas (large proportion of Sámi) with areas with a minority of Sámi residents in the same county, the prevalence of poor self-rated oral health was higher among participants in Sámi core areas compared with participants in areas with minor Sámi residents (34% vs. 25%) [102]. 

The dental service provision model in Norway is a mixture of public and private. All Norwegians up to the age of 19 years receive dental care free-of-charge. Public dental care is also available for certain high-risk groups including the mentally unwell, older persons, those with a disability or chronic condition, and the incarcerated. Most dental care to adults residing in core Sámi areas is provided through the public dental sector. A recently established dental school in Tromsø, Troms and Finnmark County (county with one-quarter of its population Sámi) has substantially improved access to culturally-appropriate dental care for Sámi in this region [103].

### 2.6. How Neoliberalism Influences Health

The socio-economic context in which people live their lives influences all aspects of health, including oral health [104]. The corporate determinants of health also play a substantial role, for example, the promotion and marketing of alcohol, tobacco, sugar-sweetened beverages and cariogenic/high fat foods [105]. The actions of global corporations, which take place in the context of powerful neoliberal ideologies and policies of most global economies, not only regulate the pricing, availability and promotion of consumables, but shape societal acceptance of consumption/use of such goods [106]. The corporate determinants of health have been defined by West and Martinue as ‘the factors that influence health which stem from the profit motive’ [107]. At an individual level, these factors include choice, agency, health and consumer-related behavior. At a broader societal-level, the factors include the political economy of globalisation and the increasingly global consumer society. These micro- and macro-level domains all flourish in, indeed cannot occur without, the neoliberalism environment. 

### 2.7. How Neoliberalism Impacts Indigenous Oral Health Inequalities

Indigenous populations in the five countries in this study carry a disproportionate burden of oral health inequalities, with the possible exception of the Sámi people in Norway. We contend that neoliberalism has overwhelmingly contributed to these inequities in five main ways: (1) increased disparities in wealth; (2) increased dominance of transnational corporations; (3) privatisation of health; (4) the neoliberal emphasis on personal responsibility and; (5) influences on systemic racism.

*Increased Disparities in Wealth*: Neoliberalism contributes to greater wealth inequities. This disproportionately impacts Indigenous populations who, irrespective of country (with the exception of Norway), are massively over-represented in lower income, under- and unemployment, and abject poverty statistics. Neoliberalism’s emphasis on ‘the self, for the self’ denies Indigenous holistic views of community sharing, participation and ownership; qualities that lead to strong social cohesion but which are cannot succeed in an environment of competition and global marketization. 

*Increased Dominance of Transnational Corporations Without Adequate Regulation or Oversight*: Transnational corporations’ products and marketing manifestly impact the oral health of Indigenous populations. Examples include the tobacco and sugar industries. Tobacco smoking rates are 70% in some Indigenous Australian communities, including among children as young as 8 years [108]. In a national survey in the United States, the highest prevalence of nicotine dependence was observed among the AI/AN population, at 64%. The second highest prevalence, 54%, was observed among the White population [109]. In Canada, 63% of the total Inuit Nunangat population aged 15 or older were daily cigarette smokers [110]. Over half (54%) of First Nations adults living on reserves and in Northern communities smoked cigarettes, with two-fifths of these individuals smoking on average 11.6 cigarettes daily [48]. This is compared to a prevalence of 15% of smokers in the general Canadian population, with only 10.8% reporting smoking daily [111]. Consumption of sugar-sweetened beverages (SSBs) in Indigenous communities in Australia, Canada, Aotearoa/New Zealand and the United States is both high and normalised, with evidence suggesting that SSB consumption in these populations is likely to increase without legislation to limit it [112]. Many Indigenous people do not trust governments with respect to safety of drinking water, which is frequently perceived to be undrinkable. In the United States, AI/AN populations have the lowest rates of indoor plumbing, with the Environmental Protection Agency stating that unregulated drinking water sources are the greatest public health risk on the Navajo Nation (due to high rates of uranium in the water) [113]. Because of the high prevalence of fracking on United States reservations, arsenic and other toxins have travelled via ground water to drinking water sources [114]. In Canada, waterborne infections are more common in Indigenous communities, with gastrointestinal infections, skin problems and birth defects frequently reported [115]. The same review reported concerns for mercury, lead, arsenic and toxic pollutants in First Nation community drinking water. Despite a 2015 election campaign promise to address the Indigenous long-term drinking water crisis in First Nations reserves by March 2021, there remain 149 drinking water advisories (including short-term advisories, and not including all First Nation communities) as of February, 2020 [116]. Freely available and affordable SSBs thus become the beverages of choice for many Indigenous persons for whom safe drinking water is simply not available. Norway is one of the countries with the highest intake of sugar and sweetened beverages, with dietary surveys showing that children and adolescents have significantly higher intakes of sugar than recommended [117]. The increased dominance of mining companies in Norway, and the consequent loss of areas for reindeer herding has had substantial impacts on the mental health of the Sámi people. The impacts of poor mental health on poor oral health among Indigenous populations is well documented [118].

*Privatization of Health*: Under the guise of free markets, powerful groups can influence access to dental care through promotion of specific dental service models. The US Department of Health and Human Services described how 36,000 Oglala Lakota in the United States were being serviced by only nine dentists [119]. In the Navajo nation, there are 22 dental clinics serving 225,639 individuals, with a dentist to population ratio of 32.3 per 100,000 [39]. Different models of dental service provision for Indigenous groups have caused conflict between various stakeholder groups, for example Alaskan Native dental therapists (who are able to provide basic dental and preventive services for cheaper than traditional services) and the American Dental Association (ADA), which publicly claims to advocate for the oral health of all. Due to perceived threats against private dentistry, the ADA filed lawsuits against the Alaskan Native Tribal Health Consortium and each of the Alaskan Native dental therapists (who, at that time, were still training), and threatened both academic institutions and American Indian organisations with a loss of donations if they became involved with the Consortium [41]. The negative effects of neoliberalism are thus exacerbated through increased emphasis on privatisation and shifts away from State welfare provision. A major difference can be seen in Norway, where, in core Sámi areas in Northern Norway, all Sámi patients have their right to speak Sámi at public dental offices. Almost all public dental clinics in this region have one or more Sámi-speaking dental health personnel. In other areas in Norway, however, adults are not prioritized in the public dental health services, meaning they predominantly need to seek care through the private market. Private dental health services in Norway do not have Sámi speaking oral health service providers, and this then impacts on the dental care utilisation of the Sámi group not eligible for public care. 

*Domination of Concepts of Personal Autonomy and Responsibility*: Indigenous populations are collectivistic and have higher rates of external locus of control and responsibility due in large part to colonisation and oppression. However, neoliberal ideology promotes personal autonomy and responsibility, often resulting in classist social derision of the lifestyles, purchasing decisions and subsequent health outcomes of the socially disadvantaged [3]. Neoliberal ideology blames individuals for their circumstances thus ignoring the greater influence of the historical and political influences on wealth distribution and other social determinants of health [3]. One example related to poverty is the shame some Aotearoa/New Zealand Māori feel about inability to pay dental bills [120]. The exploitation of these labels, and the labelling of difference, can be effectively used to create stigma through discrimination and exclusion, rejection and systemic disapproval. A newly emerging, but increasingly recognized determinant of health, stigma has profound impacts on social inequality and life chances, with manifest consequences on Indigenous oral health inequalities such as not applying for jobs because of embarrassment about missing teeth or not seeking dental care because of fear of dentists’ moral judgement [121].

*Influences on Systemic Racism*: Systemic racism is directly influenced by neoliberal policies, which by nature promote competition and supports groups in power [122]. Systemic racism has been defined as a covert form of racism expressed in the practice of social and political institutions [123,124]. It originates in the operation of established societal forces, which are increasingly neoliberal in the five countries highlighted in this paper, and therefore receives less public condemnation than racism which is more individual-level [125]. Systemic racism has unquantifiable impacts on educational attainment, political power and agency, health care, housing, employment, criminal justice, income and wealth, irrespective of country [126]. Racism has a manifest impact on oral health inequities [127], with its impacts on Indigenous oral health inequalities having been empirically examined [128,129]. A brief encapsulation of the arguments described above are presented in Table 1.

## 3. Discussion

The United States, Canada, Australia, Aotearoa/New Zealand and Norway share many similarities. All five countries have similar legal bases, with broadly similar economic systems as mixed-market, capital-based economies. The post-World War II development of a welfare state in each country was largely based on similar social policy priorities. The countries also share broadly common health system characteristics, such as universal access to health care for the socially disadvantaged, although this varies widely regarding population coverage. However, the countries differ dramatically with respect to their histories of colonisation, Indigenous reconciliation, uptake of neoliberal policies and dental health service utilisation. Although the 1763 Royal Proclamation in Canada prevented colonists from directly dealing with the Indigenous population for land, it established the precedent for subsequent treaty and land rights claims [130,131]. The 1840 Treaty of Waitangi in Aotearoa/New Zealand guaranteed indigenous Māori citizenship rights. This was followed, in 1863, by political rights through legislation and, in the 1920s and 30s, limited social welfare rights [11]. Australia, in stark contrast, followed the doctrine of *terra nullius* (land legally deemed as being unoccupied or uninhabited). This led, in turn, to nation-level dispossession of Indigenous lands and persistent refusal by the settler colonies to engage equally with the country’s First Peoples.

The 1967 Referendum in Australia was a vote to change the Constitution to give the federal parliament the power to make laws in relation to Indigenous Australians, and to allow for Indigenous Australians to be included in the census. An example of how social welfare programs exacerbate the negative impacts of neoliberal policies can be demonstrated by the different outcomes in the United States (strong neoliberal) and Canada (moderate neoliberal) from the 1980s to 2008 [132]. Canada generally showed less health inequalities due to differences in: (1) income inequality, itself resulting from differences in labour market and tax policies; (2) equality in provision of social services including health care and education, and; (3) the extent of social cohesiveness across race/ethnic- and class-based groups, including the Indigenous populations of each country [132].In Norway, the first Sámi parliament was elected in 1989, an Indigenous parliament dealing with all matters concerning the Sámi people. The Finnmark Act was passed in the Norwegian parliament in 2005. This gave the Finnmark Provincial council and the Sámi parliament joint responsibility of administering land areas which were previously deemed to be state property [133]. These areas comprise 96% of the provincial region and had always been used primarily by the Sámi. Now they belong officially, not to the Norwegian state, but to the people of the province, irrespective of Norwegian or Sámi identity [133].

Some of our findings are no doubt a reflection of health system differences in each country. However, they also reflect considerable differences in the neoliberal influences on the social determinants of health/oral health among those societies. These, in turn, reflect inequities in the distribution of experiences of discrimination, social and material security, jobs and income, and educational attainment for Indigenous and non-Indigenous groups. It is important to bear in mind that the Indigenous groups reflect a small proportion of total country population, with a scarcity of Indigenous doctors, dentists and health care workers, along with funding inadequacies, recognized in each country [134]. The possible exception is Norway, where there are no differences in funding between both the provision of health services and health service training for Indigenous and non-Indigenous; the Sámi are equal to the Norwegian population more broadly. 

In all five countries, Indigenous people rely heavily on government funding sources for health care. Empowerment is an important influence on health, and Indigenous aspirations of greater self-governance have been reflected in changes to the health care systems in all five countries, but with varying degrees of success. Whilst personal empowerment and self-determination notionally reflect neoliberal tenets of individual responsibility for health and health-related behaviours, the pervasive social and commercial determinants of health that impact on the day-to-day lives of all peoples, frequently override the positive contributions of personal empowerment. Using dental service provision as just one example, Indigenous persons throughout the world may well be empowered to seek timely and appropriate dental care, but face unique challenges in many Indigenous health centres not providing dental care, or dental care which is not culturally sensitive, or being unable to afford or attract appropriate dental personnel. This may be compounded by additional geographic and logistical factors, for example, extreme remoteness of Inuit communities in Canada and difficulties with transport in the winter months, and extreme heat in Australia. While the process of colonisation in all five countries differed, one common feature until the mid-to-late 20th century was the substantial restrictions on Indigenous people’s civil rights [11]. The exception is possibly Norway who, in recent decades, have granted Sámi legislative and governance powers which substantially improves this group’s cultural, education and health empowerment, with consequent improvements in almost all economic, educational and health outcomes.

Thus, neoliberalism not only impacts Indigenous oral health globally through policies structuring social resources, but also through more insidious psychosocial processes and constructs of shame [135]. These judgments clearly reflect the neoliberal tenets of personal responsibility and agency, personal freedom and autonomy. Reducing inequalities in Indigenous oral health at a global level requires robust policy recommendations and social change within our current global and national socio-political context: a deeper understanding of the role of neoliberalism and its problematic implications is central to this. Critically, a change in society’s perceptions that neoliberalism is inevitable is required so that a deeper, more critical and nuanced understanding of what neoliberalism actually means and how it might be used to overcome inequalities, as opposed to increasing them, is crucial. The types of social changes and policy recommendations that might facilitate this include embracing suggestions from think tanks that promote and recommend the Nordic model. There is certainly much to be learned from the Norwegian/Sámi model in terms of Indigenous autonomy, sovereignty and governance in partnership with profit-driven, multinational private corporations.

Limitations of our commentary are that only five countries were included and all authors are academics and/or dental health service providers. Our views may thus not reflect those of the wider Indigenous global population of whom we write, specifically those experiencing profound social vulnerability (the homeless, incarcerated etc). 

## 4. Conclusions

Although limited to five countries, our findings may have relevance to the broader Indigenous international setting. Almost every country has a history of both colonisation and neoliberalism, including all of Latin America and many countries in Asia. Despite important differences, we assert that the Indigenous groups share many similarities with respect to neo-colonial influences on dispossession, disrespect and despair, and that these markers of social injustice are inextricably linked with poor oral health outcomes such as those reflected in our paper. Norway was a notable exception, although it is likely that the oral and general health of Indigenous peoples in that country are still influenced by the sustained legacies of social injustices from the past.

## Figures and Tables

**Table 1:** Brief encapsulation of neoliberalism’s influence of Indigenous oral health in five countries.

	United States	Canada	Australia	Aotearoa/New Zealand	Norway
Neoliberal government	Strong	Strong	Strong	Strong	Moderate
Indigenous population as % total country population	2.2%	4.9%	3.3%	17.0%	1.3%
Separate Indigenous government/official federal policy recognizing Indigenous government	Somewhat	Yes	No	No	Yes
Indigenous oral health relative to non-Indigenous counterparts in given country	Poor	Poor	Poor	Poor	Similar
Dental service provision models for Indigenous persons	Governed through HIS *, but underfunded	Combination of private or NIHB ^†^; latter underfunded	Combination of private or public; latter long wait lists, limited services	Māori Oral Health service providers: combination of private and public, culturally-safe	Public. Dental school in county with large population Sámi; culturally-appropriate dental care

* Indian Health Service; ^†^ Non-Insured Health Benefits.
